# Transcriptome-based investigation of the response and repair mechanisms in the photosynthetic system of *Cycas panzhihuaensis* under dual high-temperature and drought stress

**DOI:** 10.1186/s12870-026-08554-2

**Published:** 2026-03-18

**Authors:** Xiaoqian Sun, Chaotian Xu, Sijing Wang, Xian Liu, Yongqiong Yang, Yanling Zheng

**Affiliations:** 1 Key Laboratory for Forest Resources Conservation and Utilizationin the Southwest Mountains of China, Ministry of Education, Southwest Forestry University, Kunming, Yunnan 650224 China; 2 Yunnan Academy of Biodiversity, Kunming, Yunnan 650224 China; 3 Key Laboratory of Forest Disaster Warning and Control Yunnan Province, Southwest Forestry University, Kunming, Yunnan 650224 China; 4 Administration Bureau of Panzhihua Cycas National Nature Reserve, Panzhihua 617000, Sichuan, China

**Keywords:** *C. panzhihuaensis*, Dual high-temperature and drought stress, Photosynthetic damage repair, LHCB gene family, Transcriptome

## Abstract

**Background:**

*Cycas panzhihuaensis*, as a national first-class protected plant, has long been threatened by dual high-temperature and drought stress. Photosynthesis is a key target of dual high-temperature and drought stress. However, the specific processes underlying post-injury recovery remain poorly characterized. Utilizing chlorophyll fluorescence parameters, chloroplast ultrastructure and transcriptome sequencing techniques, this investigation analyzes the photosynthetic damage of *C. panzhihuaensis* under dual high-temperature and drought stress and the repair mechanism of photosynthetic system after rehydration.

**Results:**

The results showed that the maximum quantum yield of photosystem II decreased by 51.2% compared with the CK., the thylakoid structure disintegrated, the osmiophilic particles increased sharply, and the photosynthetic function was seriously damaged under the combined stress. Key genetic components responsible for the function of Photosystem I and II, ATP synthase and multiple photosynthesis-related transcription factors NAC, bHLH, WRKY, MYB, and ERF were synergistically down-regulated. Rewatering repair showed a sequential strategy of ' rapid water absorption → light protection priority → structural reconstruction ': when rewatering for 3 days, the leaf relative water content showed ' overcompensation '—a transient excess water uptake beyond the pre-stress level, but the photosynthetic function was further deteriorated, and the light protection mechanism was preferentially activated; after 7 days of rewatering, the grana thylakoids overlapped, osmiophilic particles decreased, the maximum quantum yield recovered to 0.689, the core photosystem genes and transcription factors were up-regulated, and the photosynthetic function was partially restored. A key observation was the delayed recovery of the LHCB gene family, particularly LHCB1 and LHCB2 homologs.

**Supplementary Information:**

The online version contains supplementary material available at 10.1186/s12870-026-08554-2.

## Introduction

The growth and development of plants are predominantly constrained by the adverse conditions of high temperature and water deficit. Global climate change is increasing the frequency and intensity of extreme weather events, with rising temperatures and prolonged droughts posing major threats to plant growth and survival [1, 2]. These two stressors often occur simultaneously in natural environments, yet their combined effects on plants cannot be simply predicted from studies of individual stresses [3, 4]. High temperature and drought disrupt multiple physiological processes through distinct but interacting mechanisms. Heat stress impairs membrane stability and photosynthetic electron transport [5], while water deficit restricts CO₂ assimilation and promotes stomatal closure [6]. When combined, these stresses can trigger synergistic damage: for example, heat-induced stomatal opening may exacerbate water loss under drought, while drought-induced stomatal closure can limit transpirational cooling under high temperature [7]. Understanding such interactions is therefore critical for predicting plant performance under future climate scenarios. Despite progress in characterizing single-stress responses in model plants and crops, the molecular mechanisms underlying recovery from combined stress remain poorly understood. Most studies have focused on immediate stress responses, leaving a significant knowledge gap regarding how plants—particularly long-lived perennials—repair photosynthetic damage after stress relief [8, 9]. Dual high-temperature and drought stress requires a unique defense mechanism to prevent further physiological and metabolic damage [10]. The activation of differentially expressed genes is essential for tolerance [11, 12]. Dual high-temperature and drought stress can also accelerate leaf senescence, degrade chlorophyll, destroy photosynthesis mechanism [13], limit light absorption [14] and reduce productivity [15].

Photosynthesis is the core target of dual high-temperature and drought stress: high temperature destroys thylakoid membrane fluidity and inhibits electron transport chain [16]; drought reduces the stability of light-harvesting complex II (LHCII) [17] and reduces the efficiency of light energy conversion [18]. Within the thylakoid membrane system, the PSII–LHCII supercomplex is primarily localized to the appressed domains of granal stacks. In contrast, photosystem I (PS I) and ATP synthase reside in the non-appressed stroma thylakoids, which encompass the granal margins and interconnecting stromal lamellae [19, 20]. LHCII is the most abundant membrane protein complex in photosynthetic eukaryotes, accounting for about 30% of thylakoid membrane proteins [21]. Four major multiprotein complexes—Photosystem I, Photosystem II, the cytochrome b6f complex, and ATP synthase—are integral components of the thylakoid membrane [16]. The light-harvesting chlorophyll (LHC) superfamily, characterized by a conserved chlorophyll-binding domain, is classified into four distinct subgroups based on structural and functional differences. These include the canonical LHC family, the light-harvesting-like (Lil) proteins, photosystem II subunit S (PsbS), and ferredoxin-chelatase II (FCII) [22]. Within the LHC subfamily, two distinct groups, LHCA gene family and LHCB gene family, are recognized, corresponding respectively to Photosystem I and Photosystem II [23]. The light-harvesting antenna of PSI is encoded by six genes, designated LHCA1 through LHCA6 [24], and the light-harvesting antenna system of PSII is determined by a set of seven genes, namely LHCB1 to LHCB7 [17, 25, 26]. Within model organisms, the LHCB gene family exhibits one of the most rapid responses to light stimulus [27].This gene encodes an essential component of Photosystem II in plants, playing a key role in light energy capture and conversion [22] and is widely involved in various stress responses [28]. LHCB gene family -encoded proteins are core components of LHCII, which is involved in light capture, dissipation of excess energy, and membrane stacking. Altered LHCB gene family expression has been correlated with modulation of PSII antenna size and stress responses [28]. In higher plants, the LHCB gene family, LHCB1, LHCB2, and LHCB3 are responsible for encoding the principal light-harvesting proteins, which are contributes to photon capture and the regulation of photosynthetic activity [29]. The LHCB1 protein, a major constituent of LHCII, contributes to the formation and stability of grana stacks. Reduced LHCB1 levels have been associated with impaired grana structure and decreased photosynthetic efficiency in model plants [30]. Dual high-temperature and drought stress can destroy the membrane system, and the effect of combined stress is more obvious [31, 32]. While the PSII repair cycle can efficiently restore photodamaged PSII, the recovery process for photoinhibited PSI proceeds at a significantly reduced rate [33].

While the above mechanisms have been extensively characterized in angiosperm crops, far less is known about how gymnosperms—particularly relict species adapted to extreme environments—regulate photosynthetic recovery after combined stress. Gymnosperms differ fundamentally from angiosperms in several aspects relevant to stress resilience, including thylakoid membrane lipid composition, chloroplast ultrastructure organization, and stress signaling pathways [34]. Among gymnosperms, cycads represent one of the most ancient seed plant lineages, having survived major climatic shifts over evolutionary time scales [35]. Their long-lived, slow-growing nature and adaptation to resource-limited habitats suggest that they may have evolved distinct strategies for balancing stress survival and recovery—strategies that could differ markedly from those of fast-growing annual crops.

*C. panzhihuaensis* is a nationally protected plant of the highest classification in China, endemic to the dry-hot valley ecosystems within the Jinsha River basin in the country's southwestern region [36]. Within the arid-warm valley zones, yearly evaporation far surpasses annual precipitation, while peak temperatures may rise above 40 °C [36], with more heat and less precipitation [37]. Previous studies have described the individual abiotic stress responses of *C. panzhihuaensis*. In this study, Yu et al. [38] from our research group systematically compared the physiological and lipidomic responses of this species to drought, high temperature, and their combination, demonstrating that combined stress induces synergistic damage that cannot be predicted by the effects of individual stresses alone. Building on this foundation, the present study focuses specifically on the temporal repair mechanisms following combined stress, particularly the dynamics of photosynthetic recovery and the role of the LHCB gene family, which remain poorly understood [39]. At present, the temporal molecular regulatory mechanisms underlying its photosynthetic recovery—particularly the dynamic reassembly of the photosystem and the delayed repair mediated by LHCB gene family—remain to be fully elucidated. To address three central questions, this research combined analyses of chlorophyll fluorescence indices, transmission electron microscopy of chloroplast ultrastructure, and transcriptomic sequencing: molecular markers of photosynthetic damage under combined stress; the hierarchical strategy of ' light protection takes precedence over structural repair ' in the rehydration process; the mechanism of LHCB gene family recovery lag and its transcriptional regulation.

## Materials and methods

### Experimental plant specimens and applied stress treatments

Five-year-old seedlings of *C. panzhihuaensis*, supplied by the Administration of Panzhihua National Nature Reserve, were used in this study. Plants were cultivated in a greenhouse at Southwest Forestry University under a 12-h light/12-h dark photoperiod with day/night temperatures of 35/25 °C and a photosynthetic photon flux density (PPFD) of approximately 600 μmol·m⁻^2^·s⁻^1^. Twenty uniformly grown, healthy plants with stem lengths of 4–5 cm were selected. Fifteen of these plants underwent combined stress treatment: they were transferred to a growth chamber and exposed to 45 °C (day)/35 °C (night) while irrigation was withheld for five days, with PPFD maintained at the same level as the control group. The remaining 5 strains were used as control (CK). After the stress, 5 plants (D)were sampled immediately, 5 of the remaining 10 plants were sampled after three days of recovery (D3), 5 of them were sampled after 7 days of recovery (D7), and 15 stress-treated plants were transferred to normal conditions to restore normal watering. Based on the pre-experiment, sampling was conducted on the third and seventh days of rewatering, respectively.

The combined stress treatment applied in this study (5 days of water withholding at 45 °C/35 °C) was selected based on our previous characterization of synergistic damage in *C. panzhihuaensis* under drought–heat combination [38]. That study established that combined stress causes more severe photoinhibition and lipid remodeling than either stress alone. Accordingly, the present investigation focuses on the recovery phase after combined stress, without repeating single-stress controls, to specifically address the mechanisms of photosynthetic repair.

### Measurement of chlorophyll fluorescence indices

Chlorophyll fluorescence was measured using a portable PAM-2500 fluorometer (Walz, Germany) after 30 min of dark adaptation. The actinic light intensity was set at 617 μmol m⁻^2^ s⁻^1^, and the plants were allowed to acclimate for over 5 min. A saturating light pulse of approximately 8000 μmol m⁻^2^ s⁻^1^ with a duration of 0.8 s was applied. Measure the middle part of the leaves of *C. panzhihuaensis*, avoiding the leaf veins. he chlorophyll fluorescence measurements in this investigation were conducted following an established protocol previously developed by our research team [36].

### Measurement of leaf and soil relative water content

The relative water contents of both soil and leaves, abbreviated as SRWC and LRWC respectively, were quantified using a gravimetric drying technique [40], and the method of Yu Jiao [38] was used for reference. Samples of fresh soil and saturated soil (abbreviated as fw and sw) weighing two grams each were placed in an oven at 105 °C until they achieved constant mass, recorded as dw1 and dw2 respectively. Soil moisture content (SWC) and soil water retention capacity (SWHC) were calculated based on the following formulas:SWC (%) = 100% × (fw − dw1)/dw1; SWHC (%) = [(sw − dw2)/dw2] × 100%. The formula of SRWC is: SRWC = SWC/SWHC × 100%. Leaf relative water content (LRWC) was measured using a three-step gravimetric protocol. The initial fresh weight (LFW) of detached leaves was recorded first. Subsequently, leaves were fully hydrated under ambient conditions for 6 h before measuring the turgid weight (LTW). Finally, samples were oven-dried at 80 °C for 48 h to obtain the constant leaf dry weight (LDW). LRWC was then calculated as [(LFW − LDW)/(LTW − LDW)] × 100%. Five biological replicates were included for each treatment.

### Observation method of mesophyll cells and chloroplast ultrastructure

(1) Leaf samples collected from the CK, D, D3, and D7 groups were cut into approximately 1 mm pieces and primarily fixed in pre-chilled 2.5% glutaraldehyde for electron microscopic observation. The fixation was carried out at 4 °C for 24 h. Following this, the specimens were transferred into a prepared phosphate buffer and fixed for a further 4 h. (2) Rinsing: Rinsing with 0.1 M phosphoric acid for three times, 15 min each time. (3) Initial fixation was conducted with 1% osmium tetroxide for a duration of 1.5 h, after which samples were rinsed three times (15 min each) in phosphate buffer (0.1 M). (4) Dehydration: 50% acetone, 70% acetone and 90% acetone were dehydrated once, 15 min each time. Finally, 100% acetone was dehydrated once, 15 min each time. (5) Embedding: Embedding with pure acetone Epon812 resin embedding liquid (2: 1) at room temperature for 0.5 h; then they were embedded in pure acetone + Epon812 resin (1: 2) at 37 °C for 1.5 h. Finally, pure Epon812 resin embedding solution was embedded at 37 °C for 3 h. (6) Sectioning and staining: After curing at 37 °C, 45 °C and 60 °C for one day, the Reichert-Jung ULTRACUT E ultra-thin slicing machine (Austria) was used to slice at 70 nm. The sections were subjected to dual staining with uranyl acetate and lead citrate, with each staining step lasting 15 min. (7) Observation: photographed by JEM1200 electron microscope.

### RNA isolation, preparation of cDNA libraries, and high-throughput sequencing

Total RNA was isolated from eighteen samples, comprising five replicates each for the CK, D, and D3 groups, and three replicates for D7. The isolation of polyadenylated mRNA from total RNA was carried out using oligo(dT)-attached magnetic beads. Subsequent fragmentation via ion-induced shearing yielded RNA segments averaging around 300 bp in length. A fragment size of 300 bp was selected because the adapter length is fixed: shorter fragments would raise the proportion of adapter sequence, reducing usable data yield, while longer fragments hinder efficient cluster generation during sequencing. The first-strand cDNA was generated from RNA template using random hexamer primers and reverse transcriptase, followed by synthesis of the second-strand cDNA employing the first strand as the template.

After library construction, the libraries were subjected to PCR amplification and fractionated to target a specific size range of approximately 450 bp. Quality assessment was conducted using an Agilent 2100 Bioanalyzer, and both total and effective library concentrations were measured. Libraries containing unique index sequences were proportionally pooled based on their effective concentrations and the intended sequencing depth to enable multiplexed sequencing. The pooled library was uniformly diluted to a 2 nM working concentration and subsequently alkali-denatured to produce single-stranded DNA templates. Sequencing was performed on an Illumina NovaSeq 6000 platform (PE150) at Novogene Bioinformatics Technology Co., Ltd. (Beijing, China). Raw reads were filtered using Fastp v0.20.1 to remove adapter sequences and low-quality reads (Q20 threshold). Clean reads were aligned to the *C panzhihuaensis* reference genome (version 1.0) using HISAT2 v2.2.1 with default parameters. Gene expression levels were quantified as fragments per kilobase of exon per million mapped fragments (FPKM) using StringTie v2.1.5.

Differential expression analysis was conducted using DESeq2 v1.30.1 [41]. Genes with |log2FoldChange|> 1 and adjusted *P-value* < 0.05 (Benjamini–Hochberg correction) were considered significantly differentially expressed. Prior to analysis, sample quality was assessed via principal component analysis (PCA) and hierarchical clustering to verify replicate consistency. Details of the reference genomes utilized in this study are provided in Tables 1 and 2.

**Table 1:** Reference genome information

Genome	*C. panzhihuaensis* _genome_Y_chromosome.fasta
Genebuild by	https://db.cngb.org/codeplot/datasets/public_dataset?id=PwRftGHfPs5qG3gE

**Table 2:** Refers to genome annotations

Database	Number	Percentage
Swissprot	21,519	65.48
GO	13,844	42.12
KEGG	11,431	34.78
eggNOG_Category	24,487	74.51
eggNOG	26,171	79.63
ALL	32,862	100

Database is database name; Annotated: the number of genes in the species annotation file annotated in the above database; percent: The percentage of genes in this annotation file that are annotated in the above database

### qRT-PCR verification

The reliability of the RNA-Seq data was assessed by performing quantitative real-time PCR validation on 23 randomly chosen differentially expressed genes. Specific primers were designed for these genes. The primers and probe sequences used for gene detection are shown in Attachment 1. In this experiment, 1–2 pairs of corresponding primers were designed for different genes, and the *CYCAS_000540* gene (derived from *C. panzhihuaensis*) was used as the internal reference gene. The identification of gene expression level was performed by relative quantitative analysis (RQ) [42]. Total RNA was isolated from all samples with the TIANGEN RNA extraction kit. A critical procedure involved DNase I treatment to remove contaminating genomic DNA, essential for avoiding false-positive signals in subsequent qRT-PCR analyses. The purified RNA was then subjected to reverse transcription to generate first-strand cDNA for quantitative real-time PCR experiments.

After purification, RNA was reverse transcribed into complementary DNA using the TIANGEN FastKing RT Kit. To enhance reaction efficiency, the reverse transcription conditions were separately optimized for mRNA and microRNA with tailored primer designs. The resulting cDNA was diluted tenfold prior to subsequent analysis. Quantitative PCR was conducted on an ABI 7500/7900 instrument, with each sample analyzed in three technical replicates. For most genes, SYBR Green-based qRT-PCR was performed using the TIANGEN SuperReal PreMix Plus (SYBR Green) kit. For genes with low expression levels or those requiring higher specificity (including *CYCAS_002835*, *CYCAS_009550*, and *CYCAS_029248*), TaqMan probe-based qRT-PCR was employed using the TIANGEN SuperReal PreMix (Probe) kit to enhance detection sensitivity and specificity. The specificity of amplification in the SYBR Green assays was confirmed by melting curve analysis. The specificity of amplification in the SYBR Green assays was confirmed by melting curve analysis.

Data analysis was performed using two distinct strategies. For relative quantification, Ct values and relative quantities (RQ) were acquired directly from the instrumentation software, and gene expression differences were evaluated via the 2^(-ΔΔCt) approach. Absolute quantification, in contrast, relied on plasmid-derived standard curves to generate a reference model; the absolute copy number of target genes was then determined based on the corresponding copy number calculation formula.

### Transcription factor analysis

Genes defined as differentially expressed (FDR < 0.05 and |log2FC|> 1) were subjected to a query against the Plant Transcription Factor Database to identify potential transcription factors and categorize them into specific families. Subsequently, these transcription factors exhibiting altered expression were organized based on their functional roles or characteristic structural domains [43].

### Weighted gene co-expression network analysis

WGCNA analysis was performed using the R package WGCNA v1.72 with the following parameters: genes with low expression were filtered based on median absolute deviation (top 75% retained), a soft-thresholding power of β = 12 was selected to achieve scale-free topology fit R^2^ > 0.85, modules were identified using the dynamic tree cut algorithm (minimum module size = 30, deepSplit = 2, mergeCutHeight = 0.25), and modules significantly associated with leaf relative water content were defined by |r|> 0.6 and *p* < 0.05.

### Statistical analysis

All statistical analyses were performed using SPSS 27.0 (IBM Corp, USA). For chlorophyll fluorescence parameters, leaf relative water content, and soil relative water content, one-way analysis of variance (ANOVA) was conducted with treatment (CK, D, D3, D7) as the fixed factor. Prior to ANOVA, data were tested for normality (Shapiro–Wilk test) and homogeneity of variances (Levene’s test). When ANOVA indicated significant differences (*p* < 0.05), Tukey’s honestly significant difference (HSD) test was used for post-hoc multiple comparisons.

For transcriptome data analysis, differential expression was performed using DESeq2 v1.30.1. Prior to analysis, sample quality was assessed via principal component analysis (PCA) and hierarchical clustering to verify replicate consistency. Two outlier samples from the D7 group were identified and excluded based on their deviation from group clustering patterns (see RNA isolation section). DESeq2's built-in dispersion estimation and moderated fold change shrinkage effectively account for unbalanced sample sizes and heteroscedasticity, ensuring robust statistical inference despite the reduced replication in the D7 group. Genes with |log2FoldChange|> 1 and adjusted *P-value* < 0.05 were considered differentially expressed.

## Results

### Chlorophyll fluorescence parameters and relative water content of leaves and soil

**Fig. 1:**
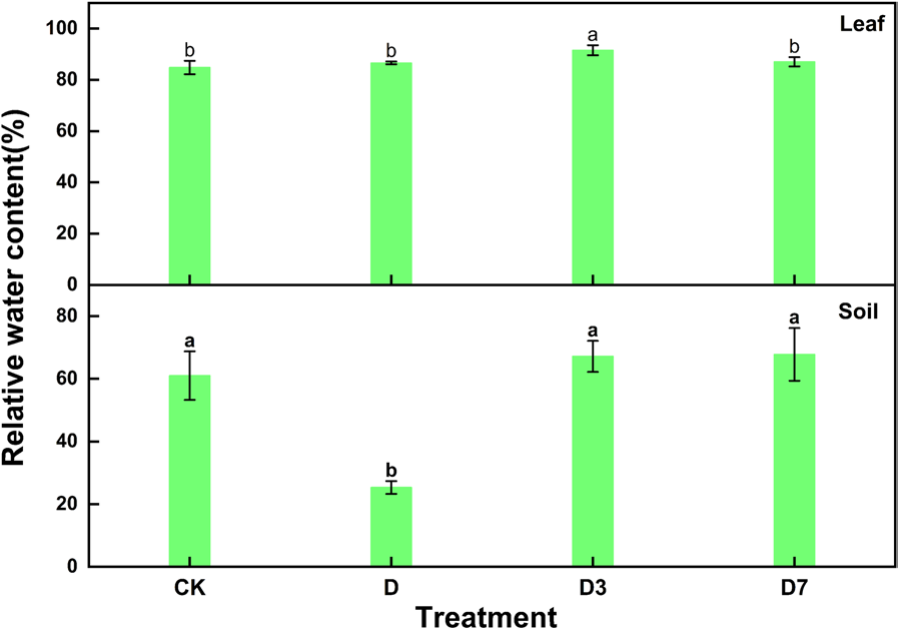
Relative water content of leaves and soil of *C. panzhihuaensis* subjected to drought and high temperature combined stress and subsequent recovery for 3D or 7D. Note: CK, control; D, 5 d of combined stress; D3, 3 d of rewatering; D7, 7 d of rewatering

The chlorophyll fluorescence parameters are shown in the original data in the article [36] (Table 1). Figure 1 presents the soil and leaf relative water content of *C. panzhihuaensis* under dual high-temperature and drought stress, followed by recovery periods of D3 or D7.

Under the dual high-temperature and drought stress, soil relative water content decreased significantly compared to the CK, while leaf relative water content remained statistically unchanged. Relative to the CK a marked decline was observed in the maximum photochemical efficiency (Fv/Fm). Meanwhile, non-photochemical quenching (NPQ) and the regulated energy dissipation component Y(NPQ) were significantly reduced, whereas non-regulated energy loss Y(NO) showed a pronounced increase. A simultaneous reduction was observed in both the actual photochemical efficiency Y(II) and the relative electron transport rate (rETR).

Under the treatment of D3, the soil moisture content after rewatering has quickly recovered to or even slightly higher than the CK level, and the relative water content of the leaves is significantly higher than the CK's 'overcompensation' phenomenon; Fv/Fm gradually increased, but still lower than CK, and the PSII structure began to repair but did not fully recover. While NPQ and Y(NPQ) showed significant increases, PSII dropped progressively, reaching a minimum value of 0.201. Conversely, qP declined to 0.437.

Under the treatment of D7, the soil moisture content was maintained fully, and the relative moisture content of leaves fell back to the level close to CK. Fv/Fm recovered to 0.689, close to CK level. Y (II) and rETR recovered to the D level, but did not reach the CK level. Y (NO) decreased to 0.430, and NPQ/Y (NPQ) maintained a high level.

### Cell ultrastructure

**Fig. 2:**
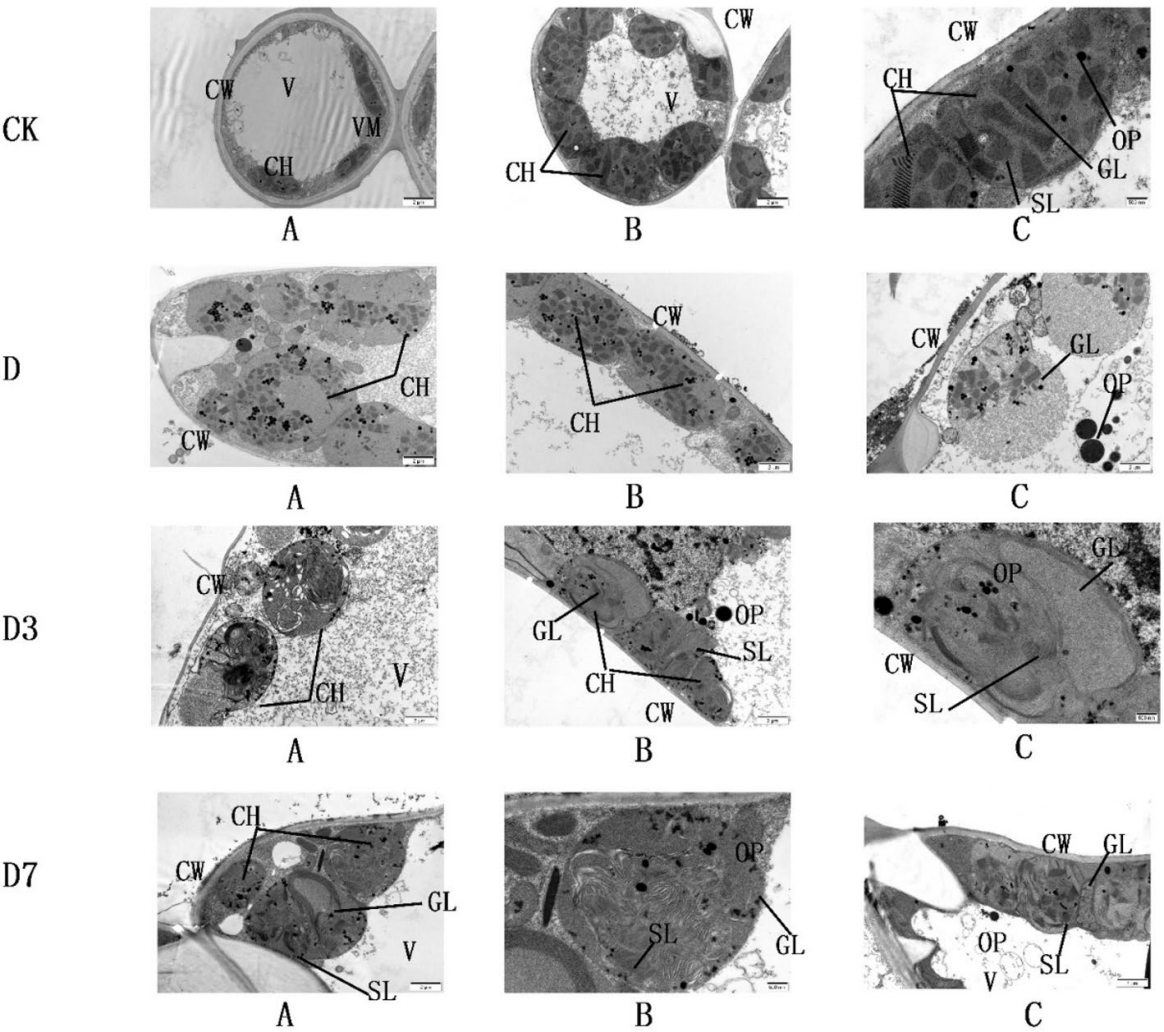
The ultrastructure of mesophyll cells and chloroplasts of *C. panzhihuaensis* under different treatments. Note: GL: grana lamella;CW: cell wall; V: vacuole; OP: osmiophilic particles;CH: Chloroplast; SL: stroma lamella. CK, control; D, 5 d of combined stress; D3, 3 d of rewatering; D7, 7 d of rewatering

Figure 2 illustrates the ultrastructural features of mesophyll cells and their chloroplasts in *C. panzhihuaensis*.

**Fig. 3:**
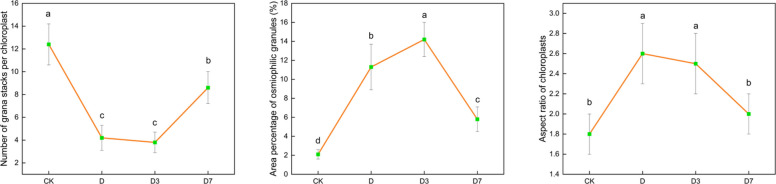
Quantitative analysis of chloroplast ultrastructural parameters under different treatments. Note: CK, control; D, 5 d of combined stress; D3, 3 d of rewatering; D7, 7 d of rewatering. Values are mean ± SD (*n* = 15–20 chloroplasts per treatment). Different letters indicate significant differences among treatments (one-way ANOVA with Tukey's HSD, *p* < 0.05)

To objectively assess ultrastructural changes, three parameters were quantified from TEM images using ImageJ software (*n* = 15–20 chloroplasts per treatment): grana stacking number per chloroplast cross-section, osmiophilic particle area (% of chloroplast area), and chloroplast aspect ratio (length/width) (Table 3; Fig. 3;).

**Table 3:** Quantitative ultrastructural parameters of chloroplasts in *C. panzhihuaensis* under different treatments

Treatment	Number of grana stacks per chloroplast	Area percentage of osmiophilic granules (%)	Aspect ratio of chloroplasts
CK	12.4 ± 1.8 a	2.1 ± 0.5 d	1.8 ± 0.2 b
D	4.2 ± 1.1 c	11.3 ± 2.4 b	2.6 ± 0.3 a
3D	3.8 ± 0.9 c	14.2 ± 2.8 a	2.5 ± 0.3 a
7D	8.6 ± 1.4 b	5.8 ± 1.3 c	2.0 ± 0.2 b

Under CK treatment, chloroplasts exhibited well-organized ultrastructure with an average of 12.4 ± 1.8 grana stacks per section, osmiophilic particles occupying 2.1% ± 0.5% of chloroplast area, and a chloroplast aspect ratio of 1.8 ± 0.2.

Under D treatment, grana stacking decreased significantly to 4.2 ± 1.1 (*p* < 0.001), representing a 66.1% reduction compared to CK. Osmiophilic particle area increased sharply to 11.3% ± 2.4% (*p* < 0.001), and chloroplast aspect ratio increased to 2.6 ± 0.3 (*p* < 0.01), indicating severe swelling and deformation.

Under D3 treatment, grana stacking remained severely reduced (3.8 ± 0.9, *p* > 0.05 vs. D), while osmiophilic particles further accumulated to 14.2% ± 2.8% (*p* < 0.05 vs. D). Chloroplast aspect ratio remained elevated at 2.5 ± 0.3.

By D7, partial recovery was evident: grana stacking increased to 8.6 ± 1.4 (*p* < 0.01 vs. D), osmiophilic particle area decreased to 5.8% ± 1.3% (*p* < 0.01 vs. D), and chloroplast aspect ratio recovered to 2.0 ± 0.2 (*p* > 0.05 vs. CK).

### Transcriptome sequencing

Transcriptome sequencing obtained 701 M raw reads. Reads containing 3'-end adapters were filtered out using Fastp, and sequences with an average base quality below the Q20 threshold were also discarded. The clean sequencing data output for each sample reached a minimum of 6 Gb. The base-call accuracy was high, with Q20 and Q30 scores attaining 98% and 96%, respectively. These metrics demonstrated reliable sequencing quality, confirming the suitability of the data for downstream analyses.

### Screening of differentially expressed genes

Gene expression differences were analyzed using DESeq, based on the following thresholds: identifying differentially expressed genes set as |log2FoldChange|> 1 and a significant *P-value* < 0.05.

**Fig. 4:**
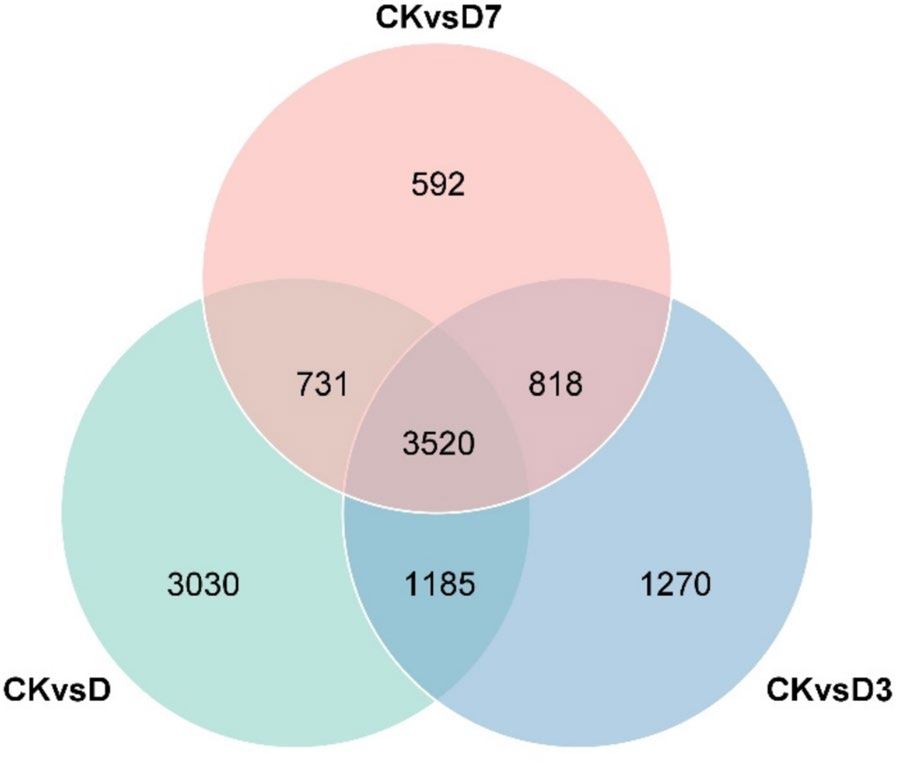
Differentially expressed gene Wayne diagram. Note: CK, control; D, 5 d of combined stress; D3, 3 d of rewatering; D7, 7 d of rewatering

Analysis of leaf gene expression levels before and after high-temperature and drought stress identified a total of 20,920 differentially expressed genes (DEGs), comprising 10,759 up-regulated and 10,161 down-regulated genes. Detailed comparisons revealed 8,466 DEGs between CK and D (4,090 up, 4,376 down), 6,793 DEGs between CK and D3 (3,598 up, 3,195 down), and 5,661 DEGs between CK and D7 (3,071 up, 2,590 down), as summarized in Fig. 4.

### GO functional enrichment analysis

**Fig. 5:**
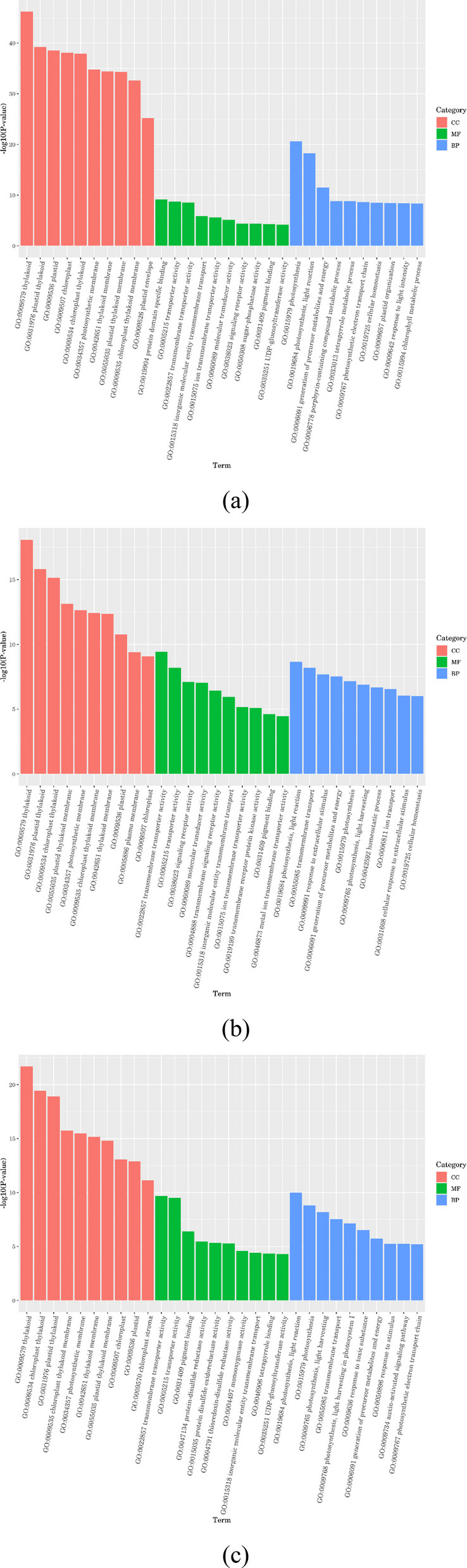
GO enrichment analysis of differentially expressed genes in *C. panzhihuaensis* under different treatments. Note: (**a**) CKvsD, (**b**) CKvsD3, (**c**) CKvsD7, FDR enrichment significance, Number is the number of genes, Category is the pathway category; the smaller the q value, the greater the enrichment factor and reference value. CK, control; D, 5 d of combined stress; D3, 3 d of rewatering; D7, 7 d of rewatering

GO enrichment analysis of the DEGs categorized the results into molecular function (MF), biological process (BP), and cellular component (CC). The ten most significantly enriched GO terms (smallest p-value) within each category are presented in Fig. 5. In CKvsD, CKvsD3 and CKvsD7 treatments, the cell process (CC) had the most DEGs, and the significantly enriched GO item was thylakoid (GO: 0009579): The flat cystic structure composed of membranes inside the chloroplast is the main place for photosynthetic light reactions (such as light energy capture, electron transport, ATP synthesis); plastid thylakoid (GO: 0031976): the thylakoid membrane system in plastids (including chloroplasts, chromoplasts, etc.), which is similar to thylakoid in function, but exists in different types of plastids. Plastid (GO: 0009536): a type of organelle with double membrane in plant and algae cells, including chloroplasts, amyloplasts, chromoplasts, etc., involved in photosynthesis, pigment synthesis, nutrient storage, etc.; chloroplast (GO: 0009507): An organelle for photosynthesis, responsible for converting light energy into chemical energy (ATP and NADPH) and participating in carbon fixation (dark reaction). Chloroplast thylakoid (GO: 0009534): specifically refers to the thylakoid structure in the chloroplast, which is the core area of photosynthetic light reaction, carrying light system I, II, ATP synthase and other protein complexes; the plastid thylakoid membrane (GO: 0055035): a lipid bilayer, hosts photosynthetic protein complexes—including PSI, PSII, cytochrome b6/f, and ATP synthase—which are integral to the conversion of light energy and electron transport processes.

### KEGG functional enrichment analysis

**Fig. 6:**
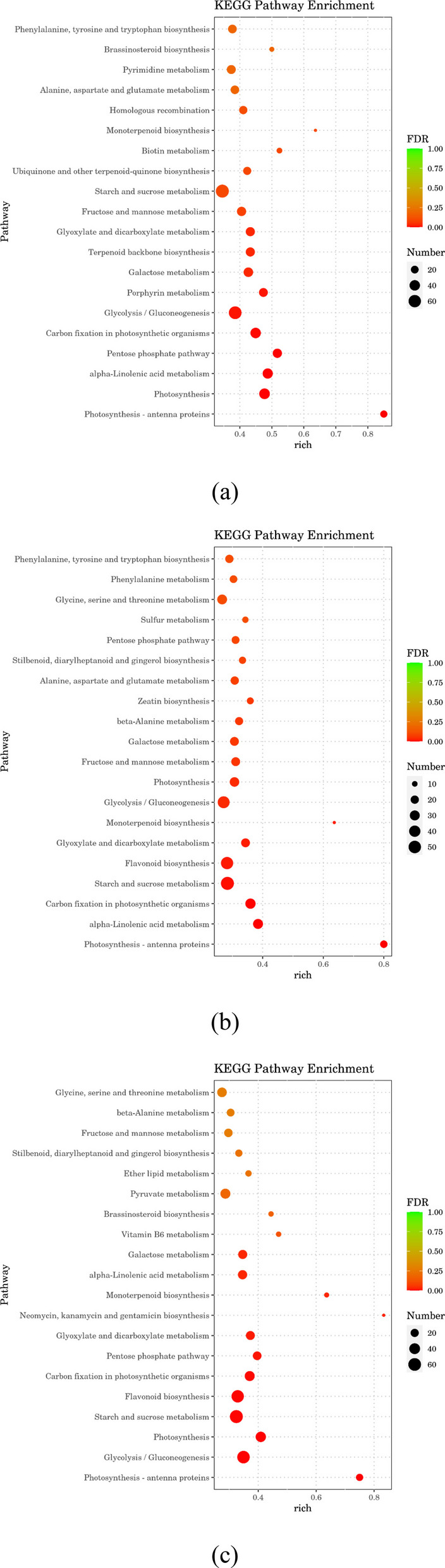
KEGG enrichment analysis of differentially expressed genes in *C. panzhihuaensis* under different treatments. Note: (**a**) CKvsD, (**b**) CKvsD3, (**c**) CKvsD7, Term is the GO enrichment entry, Richness is defined as the proportion of DEGs assigned to a specific term relative to the total number of annotated DEGs. CK, control; D, 5 d of combined stress; D3, 3 d of rewatering; D7, 7 d of rewatering

Based on the KEGG enrichment analysis of differentially expressed genes, the 20 pathways exhibiting the highest enrichment significance (lowest p-values) are presented in Fig. 6. In CKvsD, CKvsD3 and CKvsD7 treatments, the significantly enriched KEGG entries were starch and sucrose metabolism: regulation of starch synthesis and degradation, and sucrose metabolism (the main transport sugar in plants); glycolysis/gluconeogenesis: Glycolysis decomposes glucose into pyruvate and produces ATP, and gluconeogenesis is its reverse process, which synthesizes glucose from non-carbohydrate precursors; photosynthesis: including light reaction (light energy capture and conversion on thylakoid membrane) and dark reaction (carbon fixation by Calvin cycle); photosynthesis-antenna proteins: The light-harvesting complex (LHC), particularly the LHCB gene family of proteins, captures light energy and transfers it to photosynthetic reaction centers. Carbon fixation in photosynthetic organisms primarily involves the Calvin cycle, which utilizes ATP and NADPH generated during the light reactions to convert CO₂ into organic compounds. Flavonoid biosynthesis refers to the metabolic pathway responsible for producing flavonoids, a major class of secondary metabolites in plants.

### Photosynthetic pathway analysis

**Fig. 7:**
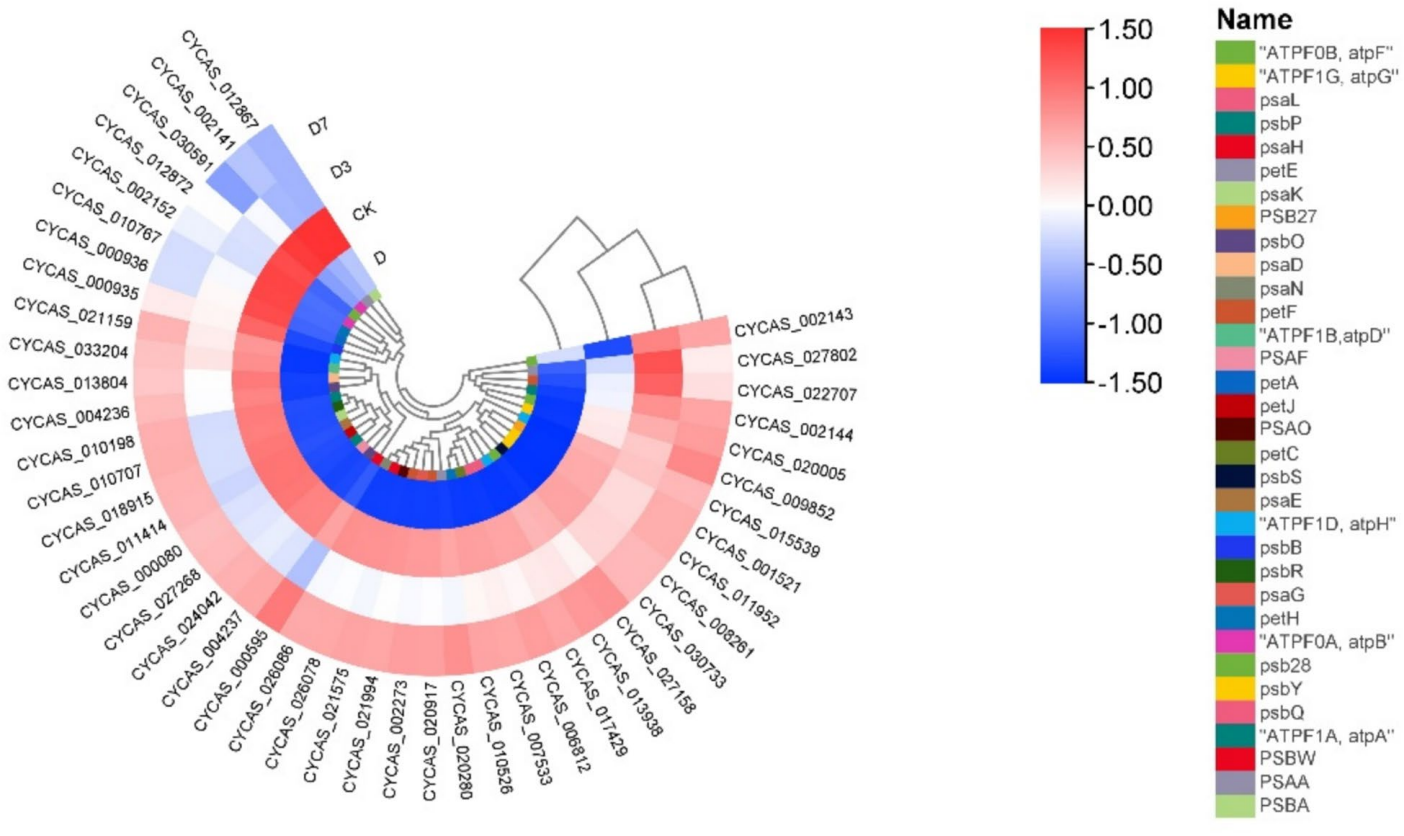
Calorimetric expression of photosynthesis pathway genes in leaves of *C. panzhihuaensis* under different treatments. Elevated expression levels are represented by red, while blue denotes lower expression. Note: Gene identifiers correspond to *C. panzhihuaensis* genome annotation. Functional annotations are provided in Attachment 2

KEGG enrichment analysis identified 45 differentially expressed genes across the D, D3, and D7 treatment groups, all annotated to photosynthesis-related pathways. Their expression patterns are displayed in the heatmap in Fig. 7, and the corresponding metabolic pathway is illustrated in Fig. 9. Compared with CK, the psa gene family encoding the PSI system under D treatment, the 10 subunits psaD, psaA, psaE, psaF, psaG, psaH, psaK, psaL, psaN and psaO of PSI were significantly down-regulated; the expression level of psa gene family was up-regulated in D3 treatment. At D7, the expression level of psa gene family gradually moved closer to CK. Eleven DEGs were identified in PSII: they were significantly down-regulated under D treatment; Under the D3 treatment condition, a marked up-regulation was observed. In contrast, the expression levels of all differentially expressed genes under D7 treatment gradually returned to levels comparable to those of the CK group. 11 DEGs of ATP synthase (F-type ATPase) system: *CYCAS_002143*, a member of ATPF0B gene family encoding F-type H^+^ -transporting ATPase subunit b, was up-regulated and the other 10 DEGs were down-regulated under D treatment. All DEGs were significantly up-regulated under D3 treatment; under D7 treatment, *CYCAS_002143*, a member of the ATPF0 B gene family encoding the F-type H^+^/Na^+^-transporting ATPase subunit b, and *CYCAS_002144*, a member of the ATPF1 A gene family encoding the F-type H^+^/Na^+^-transporting ATPase subunit α, were down-regulated, and the remaining DEGs were up-regulated. The DEGs involved in photosynthetic electron transport included petE encoding plastocyanin, petF encoding iron redox protein, petH encoding ferredoxin-coenzyme II reductase and petJ encoding cytochrome c6. Under D treatment, the petE gene family member *CYCAS_027802*, petF gene family member *CYCAS_022707* and petF gene family member *CYCAS_021994* were significantly down-regulated. All related genes were up-regulated during D3 treatment; during D7 treatment, *CYCAS_000936*, a member of the petA family encoding defatted cytochrome F, was down-regulated, and all other related genes were up-regulated.

**Fig. 8:**
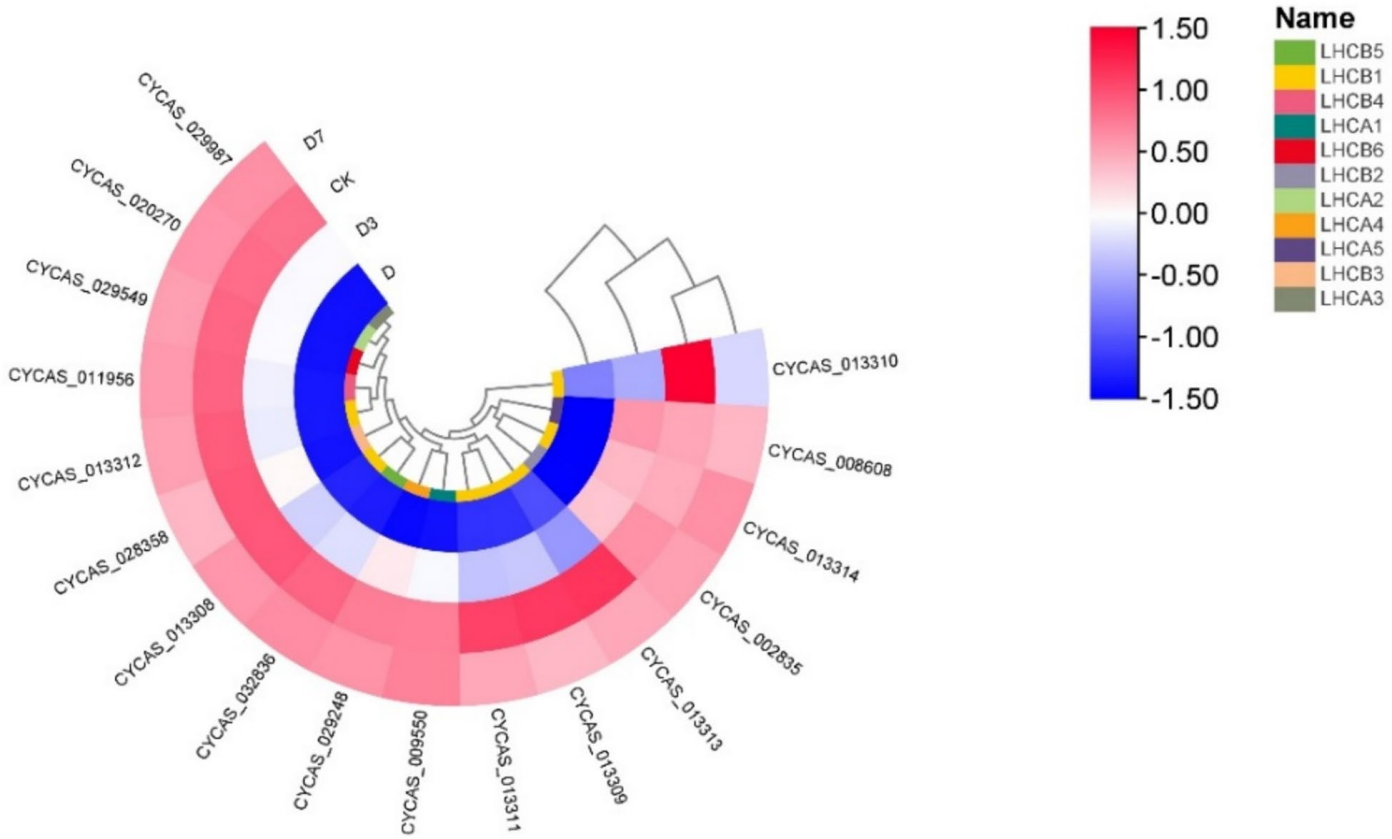
Calorimetric expression of photosynthesis-antenna protein pathway genes in leaves of *C. panzhihuaensis* under different treatments. Elevated expression levels are represented by red, while blue denotes lower expression. Note: Gene identifiers correspond to *C. panzhihuaensis* genome annotation. Functional annotations are provided in Attachment 2

**Fig. 9:**
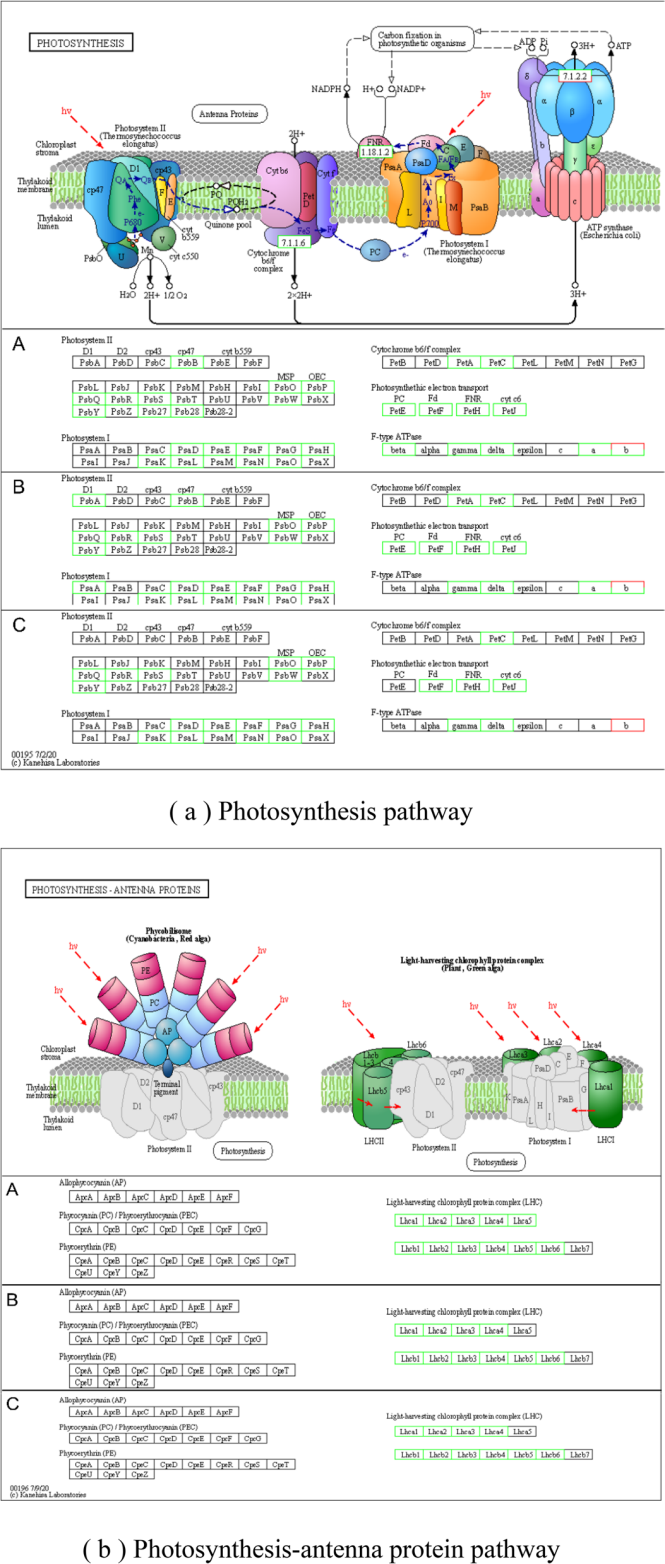
Regulation of photosynthesis and DEGs in photosynthesis-antenna protein pathway of *C. panzhihuaensis* under dual high-temperature and drought stress. Note: **A** CKvsD; **B** CKvsD3; **C** CKvsD7, red represents an upward trend in gene expression, and green represents a downward trend in gene expression. CK, control; **D**, 5 d of combined stress; D3, 3 d of rewatering; D7, 7 d of rewatering. Pathway adapted from KEGG (ko00195/ko00196); only plant-relevant components are highlighted. Components shown in gray are present in the KEGG reference pathway but are not annotated in the *C. panzhihuaensis* genome or were not detected as DEGs in this study

A set of 17 DEGs was commonly identified via KEGG enrichment analysis across the D, D3, and D7 treatment groups. All these genes were annotated to the photosynthesis pathway, with their expression heatmap presented in Fig. 8 and the associated metabolic pathway depicted in Fig. 9. Compared with CK, under D treatment, *CYCAS_009550*, *CYCAS_020270*, *CYCAS_008608*, *CYCAS_029248* and *CYCAS_029987* in the LHCA gene family encoding light-harvesting complex I chlorophyll a/b binding protein were significantly down-regulated. All genes belonging to the LHCB gene family, which encode chlorophyll a/b-binding proteins of light-harvesting complex II, exhibited a pronounced decrease in expression. During the D3 and D7 recovery phases, both the LHCA and LHCB gene family showed up-regulated expression patterns. By the D7 stage, expression of most genes had returned to levels comparable to the CK, except for CYCAS_013310 in the LHCB gene family.

### Analysis of LHCB gene family expression level in leaves of *C. panzhihuaensis* under different treatments

**Fig. 10:**
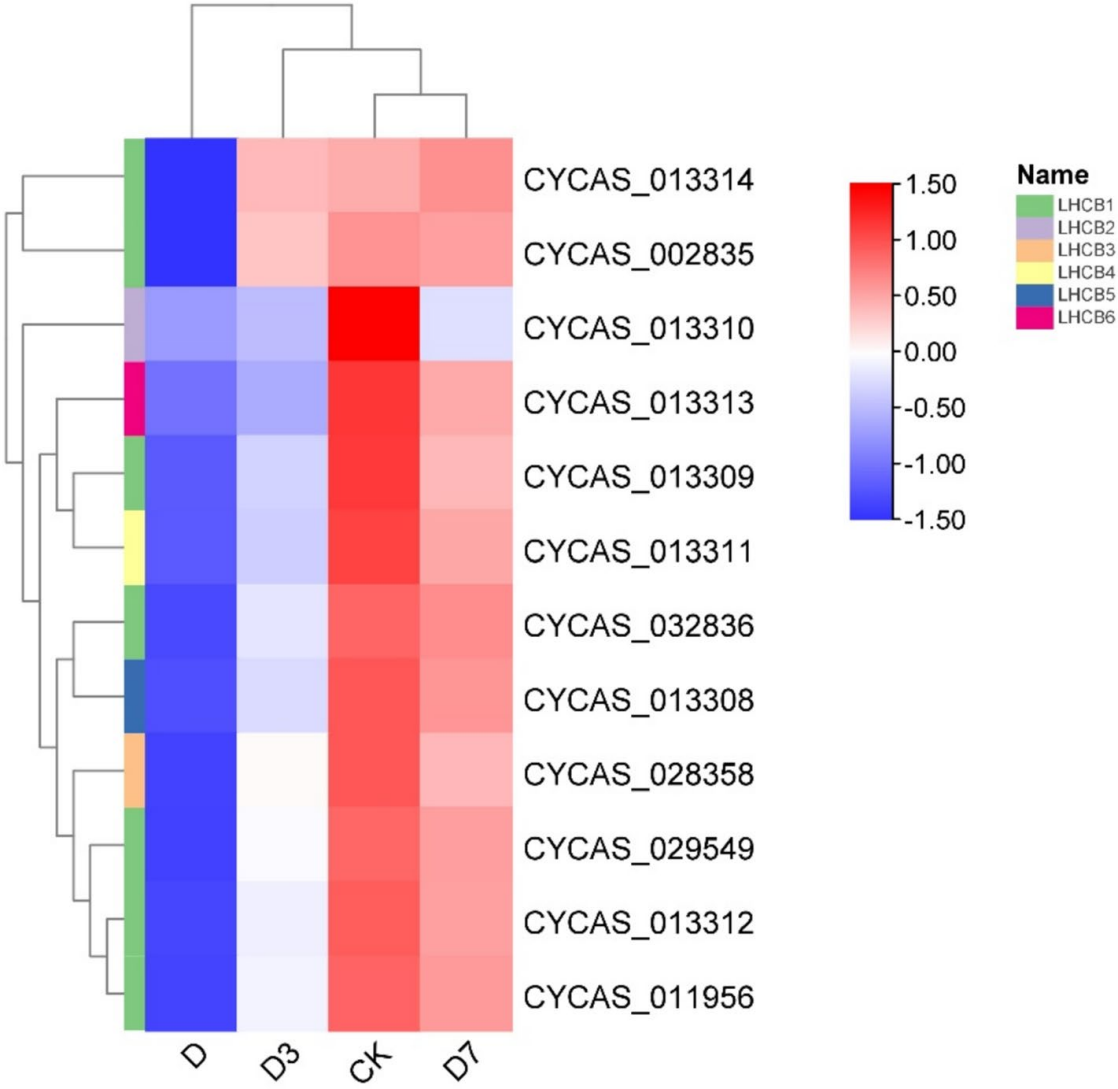
LHCB family gene clustering heat map, elevated expression levels are represented by red, while blue denotes lower expression. Note: Gene identifiers correspond to *C. panzhihuaensis* genome annotation. Functional annotations are provided in Attachment 2

As shown in Fig. 10, under D treatment, the seven genes contained in LHCB gene family showed a highly synergistic expression dynamics, and the expression levels of all LHCB1 members were significantly down-regulated. In D3 and D7 treatments, the expression of some genes such as *CYCAS_013312* and *CYCAS_013309* increased rapidly to CK level, while the recovery of genes such as *CYCAS_002835* lagged behind. The expression of *CYCAS_013310* of LHCB2 family was sharply inhibited under D treatment, > 50% down-regulated, and was still lower than CK under D7 treatment, and the repair was slow. *CYCAS_028358* of LHCB3 family was moderately down-regulated under D treatment, and returned to CK level under D3 treatment, with rapid response. *CYCAS_013311* of LHCB4 family was down-regulated by about 30% under D treatment, and the expression of D7 was higher than that of CK. *CYCAS_013308/013313* of LHCB5/6 family was significantly inhibited under D treatment, and completely recovered under D7 treatment.

### Transcription factor analysis related to photosynthetic pathway

**Fig. 11:**
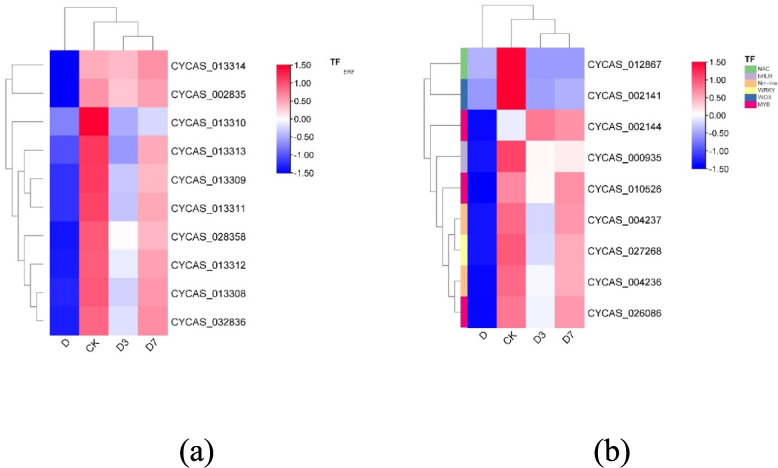
Transcription factor clustering heat map of photosynthesis-related pathways. Note: **a** Transcription factor heat map of photosynthesis-antenna protein pathway; **b** Transcription factor heat map of photosynthesis pathway. Elevated expression levels are represented by red, while blue denotes lower expression. Gene identifiers correspond to *C. panzhihuaensis* genome annotation. Functional annotations are provided in Attachment 2

From the differentially expressed genes selected within the photosynthetic pathway, a total of nine transcription factors were detected. These transcription factors are mainly attributed to the NAC, bHLH, Nin-like, WRKY, WOX, and MYB gene families. Screening of differentially expressed genes within the photosynthetic antenna protein pathway yielded 10 transcription factors. These transcription factors belong to the ERF gene family, and their transcription factor clustering heat map is shown in Fig. 11. These 19 transcription factors were down-regulated in CKvsD, CKvsD3 and CKvsD7 treatments, and up-regulated in DvsD3 and DvsD7 treatments.

### WGCNA identified a dual high-temperature and drought stress response gene module in *C. panzhihuaensis* revoluta

**Fig. 12:**
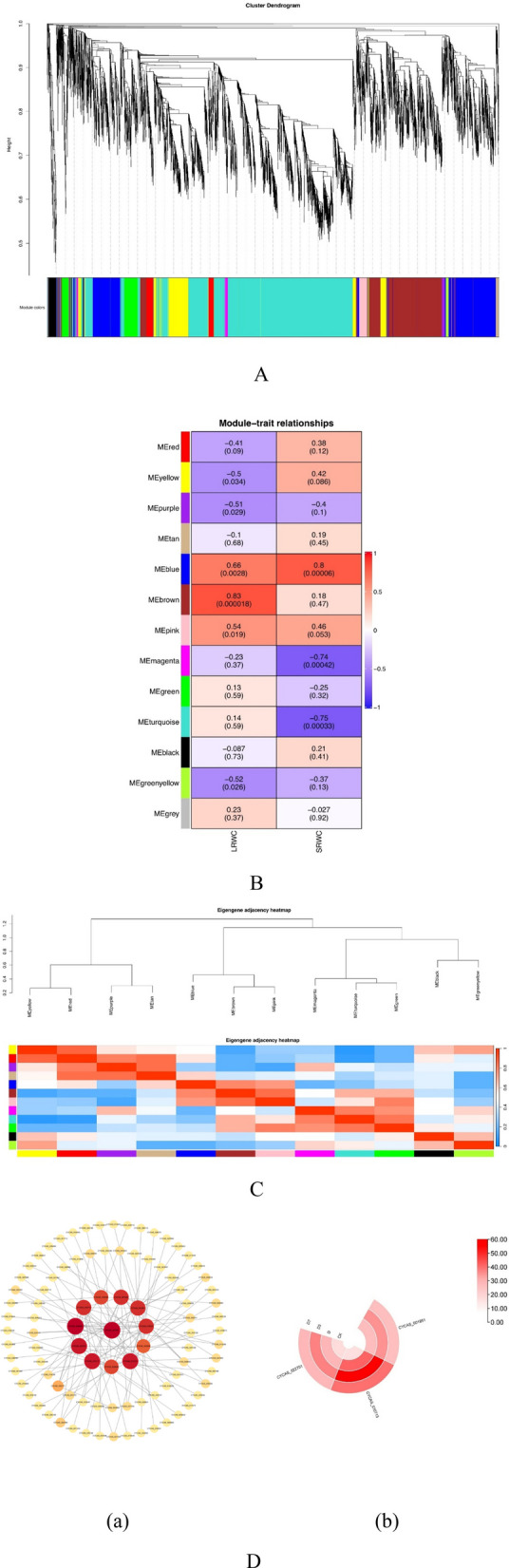
Modules and regulatory networks identified in this study. Note: **A** Modules identified by WGCNA (weighted gene co-expression network analysis). Different colors represent different modules. **B** Correlation index and correlation between phenotypic traits and modules. **C** Heat map of module connectivity. D. **a** The regulatory network of hub genes in the MEbrown module. **b** hub gene expression heat map, the deeper the color, the higher the expression level. Elevated expression levels are represented by red, while blue denotes lower expression. Gene identifiers correspond to *C. panzhihuaensis* genome annotation. Functional annotations are provided in Attachment 2

In this study, in order to investigate the transcriptional regulatory network of *C. panzhihuaensis* under the dual high-temperature and drought stress, WGCNA was used to identify specific genes highly related to target genes. Under D processing, a total of 11 modules are obtained, as shown in Fig. 12C. Analysis of trait-module associations revealed that the MEbrown module demonstrated the strongest positive correlation with the leaf relative water content under dual high-temperature and drought stress (Fig. 12B). The hub gene *CYCAS_001951* interacted with *CYCAS_010713*, *CYCAS_022701* and other genes. *CYCAS_010713* and *CYCAS_022701* mainly control protein processing and glycan synthesis in the endoplasmic reticulum, and the expression heat map between the three is provided in Fig. 12D. The expression of hub genes *CYCAS_001951*, *CYCAS_010713* and *CYCAS_022701* were low in CK, increased significantly after D treatment, and decreased gradually after D and D7 treatment.

### qRT-PCR analysis verified the transcriptome sequencing results

**Fig. 13:**
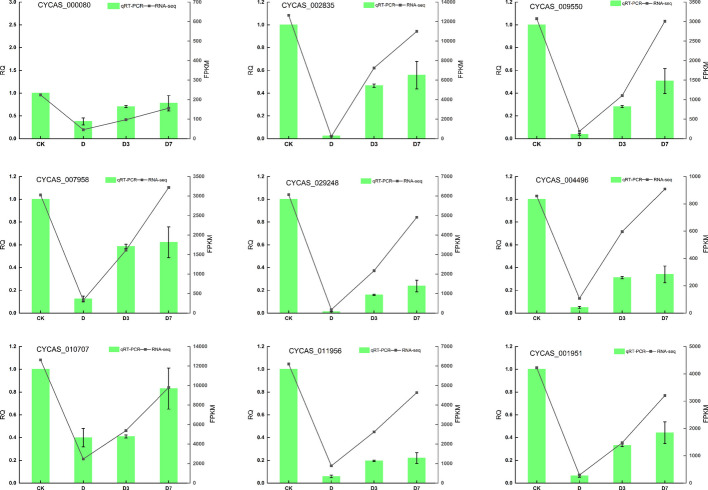
Trend of qRT-PCR and RNA sequencing results. Note: Gene identifiers correspond to *C. panzhihuaensis* genome annotation. Functional annotations are provided in Attachment 2

To validate the RNA-Seq findings, eight DEGs and associated hub genes involved in photosynthetic and antenna protein pathways were selected from the 23 qRT-PCR-verified genes for presentation, and their correlation with sequencing data was assessed (Fig. 13). Quantitative analysis revealed strong positive correlations between RNA-Seq and qRT-PCR expression patterns across all nine genes, with Pearson correlation coefficients (R^2^) ranging from 0.81 to 0.94 (all *p* < 0.01. The strongest correlations were observed for *CYCAS_002835* (R^2^ = 0.94), *CYCAS_009550* (R^2^ = 0.91), and *CYCAS_029248* (R^2^ = 0.89). These nine genes were markedly suppressed under dual high-temperature and drought stress, suggesting their potential functional roles in the plant's integrated response to dual stress conditions. Notably, the strongest suppression was observed for *CYCAS_002835*, *CYCAS_009550*, and *CYCAS_029248*, indicating their particular importance in mediating adaptation to simultaneous heat and drought. Variations in the degree of repression across these genes point to distinct functional contributions to stress adaptation. Importantly, the strong correlation between RNA-Seq and independent qRT-PCR measurements validates the reliability of the transcriptomic dataset.

## Discussion

In this study, the thylakoid disassembly induced by combined stress coincided temporally with the marked declines in Fv/Fm and Y(II). This structure–function covariation is consistent with observations in crops such as wheat and rice under drought and heat stress [44, 45]. However, unlike annual crops, which typically exhibit rapid photosynthetic recovery upon rewatering, *C. panzhihuaensis* showed further deterioration in photosynthetic function at three days after rewatering (Y(II) reached its minimum), indicating a delayed onset of repair. This delay may reflect fundamental differences in resource allocation strategies between perennial woody plants and annual herbaceous plants.

In rice, the resynthesis and assembly of the PSII core protein D1 are completed within three days after relief from heat stress [46]. In the present study, however, Y(II) and qP in *C. panzhihuaensis* decreased to their minimum values at three days after rewatering and only partially recovered to pre-stress levels by seven days. This delay may reflect fundamental differences in resource allocation strategies between perennial woody plants and annual herbaceous plants: perennials tend to prioritize survival (e.g., through sustained thermal dissipation) over immediate recovery of photosynthetic production, thereby avoiding the investment of resources in uncertain environments. Notably, the recovery timeline of *C. panzhihuaensis* resembles that of another woody plant, *Populus yunnanensis*, after drought–rewatering [47]. Suggesting that woody plants may share a conserved 'slow-recovery' strategy. The ecological significance of this strategy lies in its adaptation to the dry–hot valley environment, where dramatic fluctuations in water availability and temperature occur: delaying photosynthetic recovery prevents the initiation of energy-intensive anabolic processes before environmental conditions are fully favorable.

The progressive disintegration of grana thylakoids and the accumulation of osmiophilic inclusions are consistent with severe oxidative stress, suggesting that antioxidant systems may have been overwhelmed or insufficiently activated [48]. The significant increase in Y(NO) indicates a higher proportion of non-regulated energy dissipation, which typically reflects impaired photochemical function or a state where photoprotective mechanisms are insufficient to dissipate excess light energy. At D7, the leaf relative water content returned to the normal range. Meanwhile, photosynthetic function indices—Y(II), qP, and rETR—had essentially recovered to pre-stress levels, and Fv/Fm was significantly improved. These observations reflected the gradual repair of the photosynthetic mechanism and the progressive restoration of photochemical efficiency and electron transport capacity [49]. Key signs of structural repair in cells emerged: granal thylakoids began to re-stack, which may be related to partial reconstruction of the PSII reaction center [46]; the reduction in osmophilic granules is typically associated with controlled membrane lipid peroxidation and the gradual recovery of the electron transport chain [50]; regularization of chloroplast morphology and elimination of plasmolysis optimize the efficiency of light energy transfer [51]. Even at the D7 stage, the number of stacked thylakoids remained notably lower compared to the CK group, which may consequently impaired the light-harvesting capacity of PSII. It shows that the recovery of photosynthesis is stepwise: priority repair of thylakoid membrane system, especially PSII grana structure, and removal of oxidative damage are the prerequisites for the restart of photoreaction, but complete recovery takes longer.

The quantitative ultrastructural analysis provided objective metrics linking structural recovery to functional restoration. Grana stacking number showed a strong positive correlation with Fv/Fm across all treatments (Pearson's r = 0.89, *p* < 0.001), indicating that grana stacking density is a reliable morphological proxy for PSII functional integrity. Conversely, osmiophilic particle area correlated negatively with Y(II) (r = −0.82, *p* < 0.01), supporting the view that residual oxidative stress constrains photosynthetic recovery even after 7 days of rewatering. The incomplete restoration of grana stacking at D7 (69.4% of CK levels) paralleled the persistently low expression of LHCB family members, providing quantitative correlative evidence linking transcriptional and structural recovery.

Transcriptome analysis further elucidated the root causes of damage at the molecular level. GO analysis showed that cell components such as thylakoids (GO: 0009579) and chloroplast thylakoids (GO: 0009507) continued to be significantly enriched, which was consistent with the ultrastructure of cells and chloroplasts [52]. At D7, grana thylakoid overlap was synchronized with the up-regulation of related genes, driving partial repair of PSII [53]. KEGG analysis showed that genes of the photosynthesis pathway (ko00195) were predominantly down-regulated during stress. This transcriptional down-regulation coincided with the suppression of light-reaction activity, consistent with a role of gene expression in modulating photosynthetic capacity, accompanied by the inhibition of carbon fixation (ko00710) gene and the up-regulation of starch metabolism gene. Although genes in the photosynthetic pathway showed restorative expression, supporting electron transport resumption, the photosynthesis-antenna protein pathway (ko00196) was not markedly enriched. The photosynthesis-antenna proteins pathway (ko00196) was not significantly enriched at D7, whereas genes related to thylakoid organization (GO:0009579) continued to be overrepresented. This expression pattern may reflect a delayed transcriptional recovery of light-harvesting components [54]. The flavonoid biosynthesis pathway (ko00941) was consistently enriched under stress and during early recovery. This pattern is consistent with a role of flavonoids in mitigating oxidative stress [55]. In summary, the photoreaction repair formed by thylakoid reconstruction takes precedence over the dark reaction [56], the delayed transcriptional recovery of light-harvesting components may contribute to the incomplete restoration of photosynthetic efficiency [57]. Under the dual high-temperature and drought stress, the expression of major genes in the core pathway of photosynthesis (ko00195) was generally inhibited, including 10 psa [58] subunit genes of PSI, 11 core genes of PSII and 10 subunit genes of ATP synthase complex. The extensive down-regulation of this core photosynthetic gene expression is highly consistent with the observed structural collapse and functional collapse, which together lead to the stagnation of photosynthesis [59]. In the photosynthesis-antenna protein pathway (ko00196), some LHCA family genes showed an upward trend during the stress period, while LHCB family genes were generally down-regulated, and the balance of light energy capture and transmission was broken [60]. The persistent transcriptional lag of LHCB gene family, especially LHCB1 and LHCB2, represents a key constraint on PSII repair. Notably, while most LHCA gene family (associated with PSI) had returned to control levels by D7, LHCB gene family remained suppressed. This differential recovery pattern aligns with the asymmetric repair mechanisms of PSI and PSII: PSII repair requires de novo synthesis of D1 protein, disassembly and reassembly of supercomplexes, and involvement of multiple auxiliary factors, whereas PSI is more stable and its repair is less transcription-dependent [33, 51].

The KEGG pathway ko00941 is continuously enriched to maintain a high level of flavonoid biosynthesis, thereby responding to the threat of excess light energy and reactive oxygen species, temporarily sacrificing photosynthetic efficiency to protect photosynthetic apparatus from irreversible damage [61]. This "prioritize protection over recovery" strategy likely stems from the long-term evolutionary adaptation of *C. panzhihuaensis* to its native environment, enabling it to withstand potential subsequent stressors. Compared to previously reported crops, *C. panzhihuaensis* exhibits a distinct 'water-first, protection-next, photosynthesis-last' recovery sequence. The marked 'water overcompensation' at D3 (leaf relative water content exceeding CK) coupled with further deterioration of photosynthetic function reveals a unique stress response logic: under conditions of high water uncertainty, this species prioritizes water uptake and storage over immediate photosynthetic resumption. This strategy may represent an adaptive legacy of its evolutionary history in the drought- and heat-prone dry-hot valleys, where perennial plants must balance short-term recovery against long-term survival [37]. The persistent LHCB transcriptional lag distinguishes this gymnosperm from faster-recovering angiosperms and highlights a specific vulnerability in PSII repair that may be characteristic of relic cycads.

Photosynthesis is predominantly carried out by the integrated function of PSI, PSII, the cytochrome b6/f protein complex, the photosynthetic electron transport chain, and ATP synthase, all of which consist of pigment–protein complexes and associated light-harvesting assemblies [62]. The PSII core complex includes the inner antenna proteins CP47 (PsbB) and CP43 (PsbC), which are distinct from the peripheral light-harvesting complex II (LHCII) encoded by LHCB genes family [63]. In addition, Psb28 is an auxiliary protein involved in PSII assembly/repair, and its interaction with CP47 has been documented in cyanobacterial models [64]. Whether a similar mechanism operates in *C. panzhihuaensis* remains to be experimentally validated. Pigment-protein assemblies situated on the photosynthetic membrane mediate key light-dependent reactions, such as photon capture and electron transfer. The LHC within the plant photosystem represents a membrane-associated protein assembly that binds pigment molecules, primarily functioning in the absorption and transfer of light energy during photosynthesis [65]. Within the photosynthesis-antenna protein pathway, screened transcription factors—nine from families such as NAC [66], bHLH [67], NIN-LIKE [68], WRKY [69], WOX [70], and MYB [71], along with ten ERF [72] family members—were notably suppressed under stress (CKvsD) but exhibited increased expression during the recovery phase. This sequential expression pattern indicates that the co-activation of transcription factors is a key hub driving the repair process. At D7, chloroplast ultrastructure was reconstructed, grana thylakoids overlapped, osmiophilic particles decreased, and core photosystem functions Fv/Fm and rETR were partially restored, which was consistent with the continuous significance of ' thylakoids ' related items in GO enrichment analysis. The complete recovery of Y (II) is still limited by the continuous low expression of LHCB gene family, especially LHCB1 and LHCB2. It has been shown that transcription factors belonging to the ERF family serve as critical mediators in regulating plant adaptation to elevated temperatures [73]. The persistently low transcript levels of LHCB genes may limit the abundance of LHCII proteins, potentially contributing to the incomplete restoration of grana stacking and PSII light-harvesting capacity [74]. In this hierarchical repair strategy of ' fast water absorption → light protection priority start → thylakoid base reconstruction ', the stepwise activation of transcription factors is the key molecular basis for coordinating physiological repair and gene expression, reflecting the refined regulatory network formed by *C. panzhihuaensis* in the process of long-term adaptation to extreme environments [74].

WGCNA analysis [75] further revealed the synergistic regulation mechanism of *C. panzhihuaensis* under the dual high-temperature and drought stress from the system level. The brown module was significantly correlated with the leaf relative water content (Fig. 10B). The expression trends of *CYCAS_001951*, *CYCAS_010713* and *CYCAS_022701* were highly consistent (Fig. 11). In the early phase of stress, protein folding and modification mediated by the endoplasmic reticulum may contribute to cellular homeostasis [76]. This finding echoes the previous thylakoid structure damage and down-regulation of photosynthetic genes, indicating that the stress response is not only limited to the photosynthetic apparatus itself, but also involves a cross-organelle collaborative regulatory network. The down-regulation of this module gene expression after rehydration may be related to the recovery of cell osmotic regulation after D3 stage ' overcompensation ' water absorption, which provides transcriptional support for the ' light protection priority ' strategy.

## Conclusion

This study documented the photosynthetic responses of *C. panzhihuaensis* to a 5-day combined high-temperature and drought stress, followed by 3 and 7 days of rehydration. Stress exposure caused severe disorganization of chloroplast ultrastructure, a pronounced decline in PSII photochemical efficiency, and widespread down-regulation of genes encoding components of the photosynthetic apparatus, including PSI, PSII, ATP synthase, and light-harvesting complexes. Upon rewatering, leaf relative water content recovered rapidly, showing an overcompensation at day 3, yet photosynthetic performance continued to deteriorate, concomitant with sustained high non-photochemical quenching and further structural disruption. By day 7, partial restoration of thylakoid stacking, a reduction in osmiophilic inclusions, and increased transcript levels of most photosynthesis-related genes were observed, together with a recovery of Fv/Fm to ~ 0.689. However, the expression of several LHCB family members, particularly LHCB1 and LHCB2 homologs, remained below control levels. This transcriptional lag correlates with the incomplete recovery of Y(II) and the reduced grana stacking density observed at day 7.

Our findings highlight a pronounced asynchrony between the restoration of core photosynthetic gene expression and the recovery of light-harvesting capacity in this endangered gymnosperm. Compared to annual crops such as Arabidopsis and rice, which typically resume photosynthesis within 2–3 days of rewatering, *C. panzhihuaensis* exhibits a delayed but phased recovery strategy—prioritizing water uptake and photoprotection over immediate photosynthetic resumption—which may reflect an adaptive legacy of its evolutionary history in the drought- and heat-prone dry-hot valleys.

The persistent transcriptional lag of LHCB gene family, especially LHCB1 and LHCB2, represents a key constraint on PSII repair, distinguishing this gymnosperm from faster-recovering angiosperms. Whether this asynchrony is a general feature of long-lived plants or specific to relic cycads requires comparative studies across species. Furthermore, the hypothesized link between ERF transcription factors and LHCB gene family expression dynamics remains speculative and needs functional validation.

From a conservation perspective, these results underscore the importance of extended post-stress care for ex situ populations of *C. panzhihuaensis*: after extreme climatic events, rewatered plants should be provided with adequate time and moderate shading to allow complete photosynthetic recovery. In addition, the identified transcriptional constraints on PSII repair could inform future breeding or genetic engineering strategies aimed at improving stress resilience in crops, particularly by targeting the regulation of LHCB gene family expression or its upstream transcription factors. The brown module, correlated with leaf water content and containing ERF stress-related hub genes, provides preliminary evidence for cross-organelle coordination in stress recovery. Its functional dissection—including systematic enrichment analysis and experimental validation—represents an important direction for future research.

## Supplementary Information

Supplementary Material 1.

Supplementary Material 2.

## Data Availability

The datasets used and/or analyzed during the current study areavailable from the corresponding author on reasonable request.The raw sequencing data have been deposited in the NCBI Sequence Read Archive (SRA) under BioProject accession number PRJNA1378517（https://dataview.ncbi.nlm.nih.gov/object/PRJNA1378517）

## Abbreviations

NAC: NAM, ATAF1/2, CUC2 domain transcription factor family
bHLH: Basic helix-loop-helix transcription factor
WRKY: Transcription factors containing at least one conserved motif ‘WRKYGQK’ at the N-terminal
MYB: Myeloblastosis domain-containing transcription factor
ERF: Ethylene-responsive transcription factor
LHCB: Light-Harvesting Chlorophyll a/b-Binding Protein of Photosystem II
LHCII: Photosystem II light-harvesting complex
PSI: Photosystem I
PSII: Photosystem Ⅱ
LHC: Light-harvesting chlorophyll
Lil: Light harvesting
PsbS: Photosystem II subunit S
FCII: Errochelatase II
LHCA: Light-Harvesting Chlorophyll a/b-Binding Protein of Photosystem I
PPFD: Photosynthetic photon flux density
RNA-Seq: RNA sequencing
qRT-PCR: Quantitative real-time PCR
DEG: Differentially expressed gene
GO: Gene Ontology
KEGG: Kyoto Encyclopedia of Genes and Genomes
WGCNA: Weighted gene co-expression network analysis
NIN-LIKE: Nodule Inception-like
WOX: WUSCHEL-related homeobox
CP47: Chlorophyll Protein of 47 kDa
CP43: Chlorophyll Protein of 43 kDa
PsbA: Photosystem II subunit A
PsbC: Photosystem II subunit C
PsbD: Photosystem II subunit D
FPKM: Fragments per kilobase of the exon model per million mapped fragments
