# Supplementary material for: Transcriptome-based investigation of the response and repair mechanisms in the photosynthetic system of Cycas panzhihuaensis under dual high-temperature and drought stress
Source: BMC Plant Biol. 2026 Mar 18;26:755. doi: 10.1186/s12870-026-08554-2 (PMC13112630; doi:10.1186/s12870-026-08554-2)
Supplement: Supplementary file 1 — Supplementary Material 1. [file 12870_2026_8554_MOESM1_ESM.docx]

Attachment 1 Gene detection uses primer and probe sequences

|  | Primer sequence | |  |
| --- | --- | --- | --- |
| Gene | Forward primer e | Reverse primer | Product length /bp |
| CYCAS_013938 | ATCATTTGGCTGAGATTGC | CCATACCGAATAGTGAACCC | 112 |
| CYCAS_000080 | AGGCAAAGGGTATTGGAAC | GAATGCTGCTGTAGCCATAA | 101 |
| CYCAS_002273 | GTTTGTGTTCCTGTCCTTCC | TATTCCTTGGCTCTGGTGTC | 105 |
| CYCAS_002835 | GATGACCCTGAAGCGTTTG | GTCCTTTGCCCGTTACAATC | 112 |
| CYCAS_006812 | AAGCAGTGGCCTTATGTTCAG | TTCTTCTGGTCTTTGGGTTTG | 101 |
| CYCAS_009550 | TACCCTGGAACTGGACCTT | GAGGAATGACAACATCAGCA | 85 |
| CYCAS_009737 | ACGACAAACATTTCACCATCT | GACCGAGCATACGACCTTT | 120 |
| CYCAS_010707 | CTGCTGTGCTCCCATGTAG | AATTTCCAGTGCGGGTATT | 102 |
| CYCAS_011952 | CTGAACCAGCTCGACAGAA | GAGCCAATTCTTCTTGCACT | 127 |
| CYCAS_011956 | CGAGTTTCAGAGGAATGCTG | ATAGCCAATCGAGCGTGTT | 144 |
| CYCAS_013804 | GGACGAGACTGAGGTCCAA | GCATGTTCAAACCGACTCC | 144 |
| CYCAS_001951 | GGAGTTGAGGACCAAGGAG | CAAGATGAGCCAGCAAGTT | 119 |
| CYCAS_024042 | CGAGAACTATGGGAAACAGG | TGATACCGGGAGTGATGAA | 107 |
| CYCAS_028358 | TCAACCAGTAGCATTTCCAA | TCCATACCACAAATCTCTTCC | 129 |
| CYCAS_004496 | ATACGTTTGTGGCTGAAGTTT | GCTGAAAGAAGATGGTGGA | 125 |
| CYCAS_007533 | GCTATTTCATTACCCACTGCT | ATCATTCCCTATTGCATCCT | 114 |
| CYCAS_007958 | GCAGACAAAGAACTCAAAGGA | CATATCATCTCCCATCACCA | 126 |
| CYCAS_018915 | ATGCTGCCTGTTGTTTCAG | CCAAATCTTCCAGCAAACA | 125 |
| CYCAS_029248 | CCCGAGATACTGACCAAGG | AATGTCCTGCCATCTTCTGAT | 147 |
| CYCAS_029274 | GCTCAGACGTTTCATCCAC | GCCACCAAGTGTGCTAAGT | 133 |
| CYCAS_029549 | AAGAGAAATTGCCGAGGTTG | AATCCAATGGCTGAAAGAGG | 121 |
| CYCAS_029987 | CAATTACTGTTGGGAGGCA | TTGCTTGACTGGTTTGGTT | 111 |
| CYCAS_030733 | GAAGCAGTAACAGGAAAGGG | AGCGTGAACAGAATGAAGAAG | 104 |
| CYCAS_000540 | TCGGTCTTCCTCATCTTCC | TCTTAGTTTCCTCTTCAGCCA | 147 |
| CYCAS_000540 | TGTGATGATGAAGACGAAATG | CAAAGGCACGTTGAATAAGA | 100 |
